# The impact of intraoperative and postoperative fluid balance on complications for transthoracic esophagectomy: a retrospective analysis

**DOI:** 10.1186/s13104-023-06574-x

**Published:** 2023-11-06

**Authors:** Vered Buchholz, Riley Hazard, Zoe Yin, Nghiep Tran, Sui Wah Sean Yip, Peter Le, Benjamin Kioussis, Jake Hinton, David S Liu, Dong-Kyu Lee, Laurence Weinberg

**Affiliations:** 1 Department of Surgery, Austin Health, University of Melbourne, Melbourne, Australia; 2 Department of Anesthesia, Austin Health, Melbourne, Australia; 3 General and Gastrointestinal Surgery Research Group, The University of Melbourne, Austin Precinct, Melbourne, Australia; 4 Department of Anesthesiology and Pain Medicine, Dongguk University Ilsan Hospital, Goyang, Republic of Korea; 5 Department of Critical Care, University of Melbourne, Melbourne, Australia

**Keywords:** Esophagectomy, Cancer, Surgery, Fluid, Fluid balance, Anaesthesia

## Abstract

**Objective:**

Transthoracic esophagectomy is associated with significant morbidity and mortality. Therefore, it is imperative to optimize perioperative management and minimize complications. In this retrospective analysis, we evaluated the association between fluid balance and esophagectomy complications at a tertiary hospital in Melbourne, Australia, with a particular focus on respiratory morbidity and anastomotic leaks. Cumulative fluid balance was calculated intraoperatively, postoperatively in recovery postoperative day (POD) 0, and on POD 1 and 2. High and low fluid balance was defined as greater than or less than the median fluid balance, respectively, and postoperative surgical complications were graded using the Clavien-Dindo classification.

**Results:**

In total, 109 patients, with an average age of 64 years, were included in this study. High fluid balance on POD 0, POD1 and POD 2 was associated with a higher incidence of anastomotic leak (OR 8.59; 95%CI: 2.64-39.0). High fluid balance on POD 2 was associated with more severe complications (of any type) (OR 3.33; 95%CI: 1.4–8.26) and severe pulmonary complications (OR 3.04; 95%CI: 1.27–7.67). For every 1 L extra cumulative fluid balance in POD 1, the odds of a major complication increase by 15%, while controlling for body mass index (BMI) and American Society of Anaesthesiologists (ASA) class. The results show that higher cumulative fluid balance is associated with worsening postoperative outcomes in patients undergoing transthoracic esophagectomy. Restricted fluid balance, especially postoperatively, may mitigate the risk of postoperative complications – however prospective trials are required to establish this definitively.

**Supplementary Information:**

The online version contains supplementary material available at 10.1186/s13104-023-06574-x.

## Introduction

Despite the continuous advancement of operative techniques and perioperative care over recent years, international multicenter studies persistently demonstrate a high complication rate of up to 65% after esophagectomy. Pulmonary and gastrointestinal complications are the leading cause of morbidity, with pneumonia and anastomotic leak the most frequent complications [1–3]. Pulmonary complications and anastomotic leakage adversely affect short and long-term patient outcomes, including increased peri-operative mortality, prolonged hospital stay, readmission rate and survival [4].

Multiple factors contribute to the development of pulmonary complications and anastomotic leakage, of which with fluid overload is recognized as a substantial contributor, particularly in the context of pulmonary complications [5–7]. We conducted a retrospective analysis to evaluate the impact of intraoperative and postoperative fluid management, until postoperative day (POD) 2 on esophagectomy complications with a particular focus on respiratory morbidity and anastomotic leaks.

## Methods and materials

### Study setting

The study conducted was at a university-affiliated tertiary referral center. The protocol was registered in the Australian New Zealand Clinical Trials Registry (ACTRN234344). The study was conducted in accordance with the STROBE guidelines for observational studies [8]. This study was performed in accordance with the Declaration of Helsinki and was approved as a retrospective audit by the Human Research Ethics Committee of Austin Health (approval no Audit/45/Austin/34). The need for informed consent was waived because this was a retrospective review of hospital records, and all patient information was de-identified.

### Participants

The study evaluated patients aged 18 years or older, who underwent elective esophagectomy (three-stage, two-stage, or transhiatal esophagectomy, performed as an open, laparoscopic or hybrid procedure) between January 2010 and December 2019 for esophageal cancer, benign tumor, or end-stage motility disorders.

A standardized ERAS protocol was applied intraoperatively and postoperatively [9]. Intraoperative fluid management and vasoactive drug use were not protocolized and managed at the discretion of the attending anesthesiologist. Most patients were extubated in the theatre, and all were admitted to the intensive care unit for at least a one-night stay.

Patients’ data were extracted from the prospectively collected hospital’s electronic health records. Complications severity was graded by the Clavien-Dindo (CD) classification system [10]. In alliance with the International Esophagectomy Complications Consensus Group (ECCG) [11], complications grade CD IIIa and above were considered major complications, while complications graded CD I and II were regarded as minor. Anastomotic leaks were classified according to the ECCG definitions [11].

### Definitions

The intraoperative fluid rate was defined as mL/kg/hour of the surgical procedure. The intraoperative balance was calculated as the total volume of administrated crystalloids, colloids, and blood products minus the urine output and blood loss. Postoperative fluid balances for days 0, 1 and 2 were calculated as the cumulative volume surgery to the end of days 0, 1, and 2. High and low fluid balances were defined as above and below the median fluid balance, respectively. Postoperative complications were defined by the European Perioperative Clinical Outcome definitions [12]. Key complications of esophagectomy were defined according to the ECCG definitions [11]. Acute kidney injury (AKI) was defined per the Acute Kidney Injury Network (AKIN) classification (maximum postoperative creatinine > 1.5 preoperative creatinine) [13].

### Statistical analysis

Standard inferential statistics were used for comparing continuous and categorical variables. Univariate and multivariate analyses were performed to evaluate the contribution of risk factors to the development of complications. The statistical analysis was performed using R (version 4.2.1; 2022, R Core Team) [14]. To assess the correlation between fluid balance and complications, length of stay and mortality, first we grouped patients into high or low fluid balance groups if they were above or below the median fluid balance for each postoperative day. The approach of creating categorical variables split at the median of fluid balance was also employed in similar studies that investigated the impact of perioperative fluid balance on postoperative complications after esophagectomy [6, 15].

The multivariant analysis models included risk factors identified a-priori in the univariate analysis. POD 0, POD 1, and POD 2 were used in separate multivariate models to avoid multicollinearity. Second, we performed a univariate and multivariate regression model using cumulative fluid balance as a continuous variable. Volume was converted to liters for improved interpretability and postoperative days (POD) 0 to 2 were used in separate multivariate models to avoid multicollinearity. We analyzed the risk factors associated with major complications, major pulmonary complications, and anastomotic leaks.

Calculated odds ratios (OR) were provided with 95% confidence intervals (CIs). If required, the probability values were modified using the Bonferroni correction method. A comparative analysis is shown with the corresponding p-value and effect size. Statistical significance was defined as a two-tailed p-value < 0.05.

## Results

A total of 110 elective esophagectomies were performed during the study period. One case was excluded because of missing fluid balance data for POD 2. Thus, a total of 109 elective esophagectomies were included for analysis. Detailed patient demographic, oncological and perioperative variables are further detailed in the Additional File Table 1.

### Intra- and postoperative fluid balance and perioperative factors

**Fig. 1:**
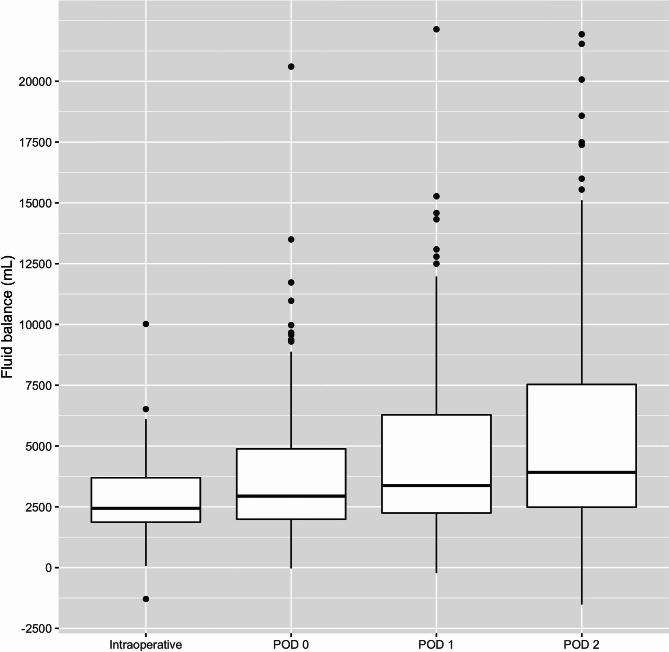
Intraoperative and postoperative cumulative fluid balances

The perioperative fluid balances during surgery to POD 2 are presented in Fig. 1. The median cumulative fluid overload was 2440 mL intraoperatively, 2940 mL on POD 0, 3372 mL on POD 1, and 3918 mL on POD 2.

We used the fluid balance on POD 2 to examine the correlation between perioperative factors and high fluid balance. High and low-volume groups were defined as above and below the median fluid balance of 3918 mL (rounded to 3950mL), respectively. Advanced clinical tumour stage T3 or T4 (*p* = 0.023) was associated with high fluid balance, while hybrid procedures were related to low POD 2 fluid balance. Patients with lower preoperative albumin did not significantly differ in their fluid balance on POD 2. Perioperative factors and their association with POD 2 fluid balance are summarized in Table 1.

**Table 1:** Perioperative fluid balances and patients’ outcomes. Data presented as number (N), proportion (%), median and interquartile range

		Intraoperative fluid balance			POD 0			POD 1			POD 2		
	All patients	Low	High	*p* Value	Low	High	*p* Value	Low	High	*p* Value	Low	High	*p* Value
Any complication													
N (%)	107 (98%)	53 (96%)	54 (100%)	0.495	52 (96%)	55 (100%)	0.243	52 (96%)	55 (100%)	0.243	53 (96%)	54 (100%)	0.495
**Anastomotic leak**													
N (%)	21 (19%)	7 (13%)	14 (26%)	0.094	5 (9%)	16 (29%)	0.014	5 (9%)	16 (29%)	0.014	3 (5%)	18 (33%)	< 0.001
**Pulmonary complication (any)**													
N (%)	34 (31%)	17 (31%)	17 (31%)	> 0.999	20 (37%)	14 (25%)	0.219	21 (39%)	13 (24%)	0.101	23 (42%)	11 (20%)	0.022
**Acute kidney injury**													
N (%)	19 (17%)	7 (13%)	12 (22%)	0.216	8 (15%)	11 (20%)	0.615	7 (13%)	12 (22%)	0.313	10 (18%)	9 (17%)	> 0.999
**Major complications (overall)**													
N (%)	46 (42%)	21 (38%)	25 (46%)	0.441	17 (31%)	29 (53%)	0.033	15 (28%)	31 (56%)	0.004	16 (29%)	30 (56%)	0.007
**Major complication per category**													
Gastrointestinal; N (%)	15 (14%)	8 (15%)	7 (13%)	> 0.999	3 (6%)	12 (22%)	0.024	3 (6%)	12 (22%)	0.024	2 (4%)	13 (24%)	0.002
Cardiovascular; N (%)	3 (3%)	0 (0%)	3 (6%)	0.118	2 (4%)	1 (2%)	0.618	2 (4%)	1 (2%)	0.618	2 (4%)	1 (2%)	> 0.999
Pulmonary; N (%)	33 (30%)	16 (29%)	17 (31%)	0.837	12 (22%)	21 (38%)	0.095	10 (19%)	23 (42%)	0.012	10 (18%)	23 (43%)	0.007
**Length of stay (days)**													
Median, (IQR)	16 (13–25)	15 (12.5–23)	19 (14-27.8)	0.058	14 (12.2–19)	22 (15–30)	< 0.001	14 (12–19)	22 (15–30)	< 0.001	14 (12–19)	22.5 (15–30)	< 0.001
**30-day mortality**													
N (%)	1 (1%)	0 (0%)	1 (2%)	0.118	1 (2%)	0 (0%)	> 0.999	1 (2%)	0 (0%)	> 0.999	1 (2%)	0 (0%)	0.618
**90-day mortality**													
N (%)	3 (3%)	0 (0%)	3 (6%)	0.495	1 (2%)	2 (4%)	0.495	1 (2%)	2 (4%)	0.495	1 (2%)	2 (4%)	> 0.999
**1-year mortality**													
N (%)	17 (16%)	3 (5%)	14/53 (26%)	0.003	5/53 (9%)	12 (22%)	0.112	5/53 (9%)	12/53 (22%)	0.112	7/54 (13%)	10/54 (19%)	0.598
**Overall mortality**													
N (%)	42/66 (64%)	16/29 (55%)	26/37 (70%)	0.303	18/34 (53%)	24/32 (75%)	0.077	16/30 (53%)	26/36 (72%)	0.131	19/32 (59%)	23/34 (68%)	0.61

### Fluid balance and outcomes

Complications were recorded in 107 patients (98%), with 46 patients (42%) experiencing major complications (CD IIIa-V). Cardiovascular, pulmonary, and gastrointestinal complications were the most common categories occurring in 84%, 71% and 52% of the patients, respectively. The single most common complication for each of those categories was hypotension (n = 67, 63%) (n = 68, 64%), pneumonia (n = 36, 33%), and anastomotic leak (n = 20, 18%).

A high intraoperative fluid balance was associated with an increased one-year mortality rate compared to patients with low fluid balance (*p* = 0.003) (Table 1); however, there was no further statistically significant correlation with length of stay, complication rate, or severity. Patients with high fluid volume balances on POD 0, POD 1, and POD 2 had a significantly prolonged median length of stay (22 days [IQR 15–30], *p* < 0.001) compared to their corresponding low-volume groups (14 days [IQR 12.2–19], *p* < 0.001). Similarly, a higher fluid balance on POD 0, POD 1 and POD 2 was associated with an increased overall incidence of major complications, major gastrointestinal complications, and anastomotic leak (Table 1).

Patients with a high fluid balance on POD 1 and POD 2 were more likely to have severe complications (of any type) (OR 3.33; 95%CI: 1.4–8.26) and severe pulmonary complications (OR 3.04; 95%CI: 1.27–7.67). There was no statistically significant difference in the incidence of kidney injury between the high and low-volume fluid balance in either of the groups.

### Multivariate analysis – complication risk factors

The risk factors associated with major complications, major pulmonary complications, and anastomotic leak are presented in Table 2. Patients were more likely to experience major complications if they had high balances on POD 0 (OR 4.46; 95% CI: 1.54–15.2), POD 1 (OR 4.15; 95% CI: 1.45–13.9), and POD2 (OR 3.33; 95% CI: 1.40–8.26). High intraoperative fluid balance was not a significant risk factor for major complications. High fluid balances on POD 1 (OR 4.15; 95% CI: 1.45–13.9) and POD 2 (OR 3.04; 95% CI: 1.27–7.67) were significant risk factors for postoperative major pulmonary compilations.

**Table 2:** Risk factors associated with major complications, major pulmonary complications and anastomotic leak

		Univariate analysis			Multivariate analysis		
Variables		OR	95% CI	p-value	OR	95% CI	p-value
**Major complications (Clavien Dindo grade > IIIa)**							
Body mass index		0.88	0.80, 0.96	0.008	0.85	0.76, 0.94	0.002
American Society of Anesthesiology class 3–4		3.80	1.58, 9.95	0.004	4.20	1.62, 11.9	0.005
Age-adjusted Charlson Comorbidity Index		1.11	0.87, 1.44	0.4			
Surgical approach (reference is the open approach)	Minimally invasive	2.84	0.26, 62.5	0.4			
	Hybrid thoracoscopy	0.89	0.25, 2.87	0.8			
High volume vs. Low volume	Intraoperative	1.40	0.65, 3.01	0.4			
	Postoperative day 0	2.43	1.12, 5.38	0.026	4.46	1.54, 15.2	0.009
	Postoperative day 1	3.36	1.53, 7.62	0.003	4.15	1.45, 13.9	0.012
	Postoperative day 2	3.05	1.40, 6.85	0.006	3.33	1.40, 8.26	0.007
Cumulative fluid balance (L)	Intraoperative	1.10	0.87, 1.41	0.4			
	Postoperative day 0	1.10	0.97, 1.26	0.14			
	Postoperative day 1	1.14	1.03, 1.28	0.016	1.15	1.03, 1.31	0.022
	Postoperative day 2	1.15	1.05, 1.26	0.003	1.16	1.05, 1.29	0.005
**Major pulmonary complications**							
Body mass index		0.95	0.86, 1.03	0.2			
American Society of Anesthesiology class 3–4		3.86	1.44, 12.3	0.012	3.46	1.25, 11.3	0.024
Age-adjusted Charlson Comorbidity Index		1.10	0.85, 1.43	0.5			
Surgical approach (reference is the open approach)	Minimally invasive	4.81	0.44, 106	0.2			
	Hybrid thoracoscopy	0.72	0.15, 2.58	0.6			
High volume vs. Low volume	Intraoperative	1.12	0.49, 2.55	0.8			
	Postoperative day 0	2.16	0.94, 5.13	0.072			
	Postoperative day 1	3.16	1.35, 7.82	0.010	4.15	1.45, 13.9	0.012
	Postoperative day 2	3.34	1.43, 8.27	0.007	3.04	1.27, 7.67	0.015
Cumulative fluid balance (L)	Intraoperative	1.24	0.97, 1.63	0.094			
	Postoperative day 0	1.16	1.02, 1.33	0.034	1.14	1.00, 1.32	0.062
	Postoperative day 1	1.17	1.05, 1.31	0.005	1.16	1.04, 1.30	0.012
	Postoperative day 2	1.16	1.07, 1.28	0.001	1.15	1.05, 1.27	0.004
**Anastomotic leak**							
Body mass index		1.08	0.99, 1.19	0.088	1.08	0.98, 1.20	0.13
American Society of Anesthesiology class 3–4		2.43	0.81, 9.00	0.14			
Age-adjusted Charlson Comorbidity Index		1.08	0.80, 1.46	0.6			
Surgical approach (reference is the open approach)	Minimally invasive	1.92	0.09, 21.1	0.6			
	Hybrid thoracoscopy	0.32	0.02, 1.79	0.3			
High volume vs. Low volume	Intraoperative	2.40	0.91, 6.87	0.086			
	Postoperative day 0	4.02	1.43, 13.2	0.012	4.46	1.54, 15.2	0.009
	Postoperative day 1	4.02	1.43, 13.2	0.012	4.15	1.45, 13.9	0.012
	Postoperative day 2	8.67	2.69, 39.0	0.001	8.59	2.64, 39.0	0.001
Cumulative fluid balance (L)	Intraoperative	1.47	1.10, 2.03	0.011	1.48	1.11, 2.03	0.01
	Postoperative day 0	1.23	1.07, 1.45	0.007	1.24	1.07, 1.46	0.006
	Postoperative day 1	1.25	1.11, 1.43	0.001	1.25	1.11, 1.43	0.001
	Postoperative day 2	1.19	1.08, 1.32	0.001	1.19	1.08, 1.32	0.001

Regarding anastomotic leaks, a high postoperative high fluid balance was an independent risk factor throughout the postoperative monitored period, with a high fluid balance on POD 2 having the most effect (OR 8.59; 95%CI: 2.64-39.0), followed by POD 0 (OR 4.46; 95%CI: 1.54–15.2) and POD 1 (OR 4.15; 95%CI: 1.45–13.9) (Table 2).

The findings for fluid balance as a continuous variable were similar to when fluid balance was used as a categorial variable i.e., patients with a high fluid balance on POD 1 and POD 2 were more likely to have severe complications of any type, severe pulmonary complications, and anastomotic leak. For every 1 L extra cumulative fluid balance in POD 1, the odds of a major complication increase by 15%, while controlling for body mass index and American Society of Anaesthesiologists (ASA) class.

## Discussion

Our data highlight the significant negative impact of daily high postoperative positive balances on complications severity, the incidence of pulmonary complications, and anastomotic leaks. The association between volume overload and overall morbidity is consistent with a growing body of literature. Eng et al. showed that high intraoperative volume resulted in more complications per patient and increased major complications [15]. Glatz et al. demonstrated a similar trajectory for postoperative volume overload, showing that fluid balance on POD 0 and the cumulative fluid balance by POD 4 were linked to increased complication severity (CD grade) [5].

Pulmonary complications, particularly pneumonia, cause substantial morbidity following esophagectomy. In the context of esophagectomy, one-lung intubation and thoracotomy-related respiratory pain are significant contributing factors. In addition, excessive fluid may add risk by inducing extravascular pulmonary tissue oedema, altering the lung physiology [16]. We demonstrated a significant correlation between volume overload on POD 1 and POD 2 and the severity of pulmonary complications. Similarly, others reported an association between the perioperative fluid balance in transthoracic esophagectomies and the number of respiratory complications [7, 17–19] or the incidence of pneumonia [5–7]. A similar correlation was not demonstrated in transhiatal esophagectomy [15], and likely reflects the reduced risk for respiratory complications using this approach.

Our findings also demonstrated a significant association between fluid excess and anastomotic leaks. Similarly, Glatz et al. reported that intra- and postoperative overload induces a three-fold incidence of anastomotic leakage. In addition, Kubo et al. showed a similar association in minimally invasive esophagectomy [5, 6]. There are several plausible explanations for our findings. First, the ECCG benchmarked rates for anastomotic leak rates are reported at 11.4%. (2). The ECCG’s low leak rate may reflect its high-volume participating canters’ expertise. Our leak rates are similar to those reported for medium-volume centers across Europe and Australia [20]. Second, our unit’s higher-risk patient selection may differ from other cohort studies published in the literature, including the ECCG. Patients with higher CCI scores and more locally advanced cancers are more likely to have higher complication rates, including anastomotic leaks. Ultimately, it is imperative to acknowledge the necessity for a more thorough investigation into strategies aimed at mitigating anastomotic leak rates. This investigation should encompass an evaluation of the selection of anastomosis techniques as well as enhancements to other facets of perioperative care.

Higher volumes of fluids were given intraoperatively compared to the postoperative daily volume. Rapid hemodynamic changes during the operation can result from analgesia, surgical maneuvers, blood losses, or one lung intubation and require fluid administration in response. Despite that, we could not demonstrate a significant adverse effect of intraoperative fluid overload on complications (increased severity, pulmonary complication, or anastomotic leak). In contrast, Eng et al. reported that high intraoperative fluid volume increased the complication rate and severity per patient but did not specifically affect pulmonary complications and anastomotic leak [15], suggesting a distributed rather than a specific system effect. Post-surgical volume overload may then be compensated in the intensive care unit. In our study, the fluid excess on the day of surgery (POD 0) was more detrimental in terms of worse outcomes than the intraoperative fluid balance, which has also been demonstrated by others [5, 7].

Our analysis showed that positive fluid balance on POD 0 to POD 2 was linked to adverse outcomes. For every 1 L extra cumulative fluid balance in POD 1, the odds of a major complication increase by 15%, even when controlling for BMI and ASA class. In other studies, examining the early postoperative period, only the POD 1 fluid balance was associated with a higher risk for pulmonary complications or anastomotic leak [6, 19]. In contrast, Glatz et al. reported that the total fluid balance by POD4, rather than the sole daily fluid quantification, was associated with poor outcomes (complication severity, pneumonia, and anastomotic leak) [5]. The literature discrepancies regarding specific critical days likely reflect the unavoidable variability in the environment of non-standardized retrospective studies, but overall highlight the need for a more protocolized and restrictive approach. Further prospective research is required to establish a protocolized approach, along with intraoperative and postoperative fluid management guidelines.

### Limitations

Fluid administration was at the discretion of either the anaesthetist in the theatre, or the surgeon, and was confined to the early postoperative period. Furthermore, we defined ‘volume overload’ arbitrarily and without correcting to patient parameters (e.g., weight). This study was conducted in a single-center hospital, which may limit its external validity to other centers and countries. Similarly, our findings are not generalizable to other types of major abdominal and thoracic surgery. We did not collect the number of hypotension epochs, nor did we quantity the magnitude of each hypotensive event. We acknowledge that hypotension is associated with microcirculatory hypoperfusion and adverse outcomes [21, 22]. Finally, because of the granular data collected, in addition to the accurate reporting and cross checking of post-esophagectomy complications and validated CD severity classification system, the complications outlined in our research can be readily compared with preexisting and future studies.

### Electronic supplementary material

Below is the link to the electronic supplementary material.

Supplementary Material 1

## Data Availability

The datasets analyzed for this study are available from the corresponding author.

## Abbreviations

AKI: Acute kidney injury
AKIN: Acute Kidney Injury Network
AJCC: American Joint Committee on Cancer
ASA: American Society of Anaesthesiologists Society
BMI: Body mass index
CCI: Charlson Comorbidity Index
CD: Clavien-Dindo
CI: Confidence interval
ERAS: Enhanced Recovery After Surgery
ECCG: Esophagectomy Complications Consensus Group
GDFT: Goal-directed fluid therapy
ECCG: International Esophagectomy Complications Consensus Group
OR: Odds ratio
POD: Post-operative day
