# Supplementary material for: The impact of intraoperative and postoperative fluid balance on complications for transthoracic esophagectomy: a retrospective analysis
Source: BMC Res Notes. 2023 Nov 6;16:315. doi: 10.1186/s13104-023-06574-x (PMC10629189; doi:10.1186/s13104-023-06574-x)
Supplement: Supplementary file 1 — Supplementary Material 1 [file 13104_2023_6574_MOESM1_ESM.docx]

**Additional File Table 1:** Detailed patient demographic, oncological and perioperative variables

| **Variables** | **All patients**  **N=109** | **POD 2 fluid balance** | | ***p*-value** |
| --- | --- | --- | --- | --- |
|  |  | **< 3950mL**  **N=55** | **≥ 3950mL**  **N=54** |  |
| **Age, years** | 64.3 ± 9.6 | 62.8 ± 9.5 | 65.8 ± 9.5 | 0.054 |
| **Gender** |  |  |  |  |
| Male, n (%) | 90 (83%) | 42 (76%) | 48 (89%) | 0.129 |
| Female n (%) | 10 (17%) | 13 (24%) | 6 (11%) |  |
| **Body mass index** (kg/m^2^) | 27.0 ± 4.9 | 26.7 ± 4.8 | 27.3 ± 5.1 | 0.687 |
| **ASA** |  |  |  |  |
| ASA I-II, n (%) | 36 (33%) | 22 (40%) | 14 (26%) | 0.154 |
| ASA III-IV, n (%) | 73 (67%) | 33 (60%) | 40 (74%) |  |
| **ACCI**, mean ± SD | 4.4 ± 1.6 | 4.3 ± 1.5 | 4.5 ± 1.6 | 0.185 |
| **Tumor location** |  |  |  |  |
| Proximal third, n (%) | 3/107 (3%) | 2/55 (4%) | 1/52 (2%) | 0.702 |
| Middle third, n (%) | 5/107 (5%) | 4 /55 (7%) | 1/52 (2%) |  |
| Distal third, n (%) | 34/107 (32%) | 18/55 (33%) | 16/52 (31%) |  |
| Gastroesophageal junction, n (%) | 58/107 (54%) | 28/55 (51%) | 30/52 (58%) |  |
| Other, n (%) | 7/107 (7%) | 3/55 (5%) | 4/52 (8%) |  |
| **Histology** |  |  |  |  |
| Adenocarcinoma, n (%) | 89 (82%) | 43 (78%) | 46 (85%) | 0.369 |
| Squamous cell carcinoma, n (%) | 13 (12%) | 9 (16%) | 4 (7%) |  |
| Other, n (%) | 7 (6%) | 3 (5%) | 4 (7%) |  |
| **cT-stage** |  |  |  |  |
| T0-2, n (%) | 16/51 (31%) | 12/26 (46%) | 4/25 (16%) | 0.034 |
| T3-4, n (%) | 35/51 (69%) | 14/26 (54%) | 21/25 (84%) |  |
| **cN-stage** |  |  |  |  |
| N0, n (%) | 64/103 (62%) | 36/53 (68%) | 28/50 (56%) | 0.524 |
| N1, n (%) | 37/103 (36%) | 16/53 (30%) | 21/50 (42%) |  |
| N2-3, n (%) | 2/103 (2%) | 1/53 (2%) | 1/50 (2%) |  |
| **Preoperative albumin (g/L)**, mean ± SD | 37.5 ± 3.6 | 37.8 ± 3.7 | 37.2 ± 3.4 | 0.153 |
| **Surgical approach** |  |  |  |  |
| Open, n (%) | 93/109 (85%) | 43 (78%) | 50 (92%) | 0.018 |
| Minimally invasive n (%) | 3/109 (3%) | 1 (2%) | 2 (4%) |  |
| Hybrid thoracoscopy n (%) | 13/109 (12%) | 11 (20%) | 2 (4%) |  |
| **Intraoperative Fluid** (mL) |  |  |  |  |
| All fluids, median (IQR) | 3,500 (2,900-5,000) | 3,100 (2,400-3,800) | 4,600 (3,425-6,000) | <0.001 |
| Crystalloid, median (IQR) | 3,000 (2,400-4,000) | 3,000 (2,000-3,075) | 4,000 (3,000-5,000) |  |
| Colloid, median (IQR) | 200 (0-500) | 200 (0-500) | 300 (100-500) |  |
| Intraoperative red blood cells, n (%) | 8 (7%) | 3 (5%) | 5 (9%) | 0.461 |
| Fluid rate, mL/kg/hr, median (IQR) | 6.1 (4.5-8.2) | 4.7 (3.9-6.6) | 7.3 (5.3-9.0) | <0.001 |
| **Vasopressors** |  |  |  |  |
| Metaraminol, n (%) | 86 (79%) | 43 (78%) | 43 (80%) | <0.001 |
| Noradrenaline, n (%) | 49 (45%) | 26 (47%) | 23 (43%) | 0.489 |
| Analgesia, epidural, n (%) | 37 (34%) | 15 (27%) | 22 (41%) | >0.999 |
| **Estimated blood loss**, mL. median (IQR) | 340 (200-500) | 300 (190-400) | 420 (275-557.5) | 0.160 |
| **Perioperative blood transfusion** n (%) | 8 (7.3%) | 3 (5.4%) | 5 (9.3%) | 0.693 |
| **Urine output**, mL median (IQR) | 580 (400-780) | 600 (428.5-800) | 500 (358.8-772.5) | 0.750 |
| **Operative time**, minutes, median (IQR) | 450 (407-495) | 455 (415.5-490) | 447.5 (401.2-510.8) 510.8) | 0.701 |

ASA, American Society of Anesthesiology; ACCI, Age-adjusted Charlson Comorbidity Index; MIO, SD, Standard Deviation
